# A universal explanation of tunneling conductance in exotic superconductors

**DOI:** 10.1038/srep31352

**Published:** 2016-08-11

**Authors:** Jongbae Hong, D. S. L. Abergel

**Affiliations:** 1 Center for Theoretical Physics of Complex Systems, Institute for Basic Science, Daejeon 305-811, Korea; 2 Nordita, KTH Royal Institute of Technology and Stockholm University, Roslagstullsbacken 23, SE-106 91 Stockholm, Sweden; 3 Center for Quantum Materials, KTH and Nordita, Roslagstullsbacken 11, SE-106 91 Stockholm, Sweden

## Abstract

A longstanding mystery in understanding cuprate superconductors is the inconsistency between the experimental data measured by scanning tunneling spectroscopy (STS) and angle-resolved photoemission spectroscopy (ARPES). In particular, the gap between prominent side peaks observed in STS is much bigger than the superconducting gap observed by ARPES measurements. Here, we reconcile the two experimental techniques by generalising a theory which was previously applied to zero-dimensional mesoscopic Kondo systems to strongly correlated two-dimensional (2D) exotic superconductors. We show that the side peaks observed in tunneling conductance measurements in all these materials have a universal origin: They are formed by coherence-mediated tunneling under bias and do not directly reflect the underlying density of states (DOS) of the sample. We obtain theoretical predictions of the tunneling conductance and the density of states of the sample simultaneously and show that for cuprate and pnictide superconductors, the extracted sample DOS is consistent with the superconducting gap measured by ARPES.

Tunneling conductance of non-strongly-correlated materials (where the electron–electron interactions do not have a fundamental effect on the physics) is simply understood because the *dI*/*dV* as a function of *V* (where *V* is the voltage difference between the sample and the tip and *I* is the measured current) is known to correspond to the DOS of the sample. However, for strongly correlated materials (SCMs) this is not true because the strong on-site interactions fundamentally change the tunneling mechanism by which electrons move from the current source to the sample itself. Nevertheless, this interpretation is frequently used when discussing SCMs, which can lead to confusing inconsistencies. For example, a sharp peak indicating the superconducting gap of cuprate superconductors is observed in the ARPES data near the nodal region^1^,^2^, but in the STS data, two prominent side peaks occur at much higher energy^3^,^4^,^5^. Also, STS data for strongly correlated pnictide superconductors display two prominent side peaks which are much bigger than the energy scale of the superconducting transition temperature^6^. Therefore, explaining the structure of tunneling conductance of SCMs is one of the most challenging and urgent subjects in condensed matter physics. This situation will remain until a comprehensive theory taking into account the non-equilibrium nature of the STS experiment and the strong electron–electron interactions has been developed.

The main purpose of this study is to prove that the two prominent side peaks in the STS data are produced by the interplay between strong electron correlations in the sample and the non-equilibrium situation imposed by the experimental setup. An additional highlight of this study, just as important as the interpretation of the side peaks, is that we can effectively find the DOS of the specific 2D SCM under study within the fitting procedure in the theory. We find that the DOS predicted for cuprate and pnictide superconductors are consistent with the data given by ARPES^2^,^7^.

The non-equilibrium calculation of *dI*/*dV* for SCMs is achieved by generalizing a theory for the non-equilibrium Kondo effect in zero-dimensional mesoscopic systems^8^,^9^ to the extended 2D situation. Some mathematical details are presented in the Methods section and in the Supplementary Materials, but in summary, the non-equilibrium tunneling dynamics are encoded within the non-equilibrium many-body Green’s function for the mediating site (MS, indicated by the subscript “*d*”), *ρ*_*d*_(*ω*), which is derived from the Liouville operator. Once this is known, the tunneling conductance can be computed from the formula in equation (2). The advantage of using the Liouvillian approach instead of the Hamiltonian approach is the availability of a complete set of basis vectors, which is not possible in the latter case. The difficulty with the Liouvillian approach arises in the calculation of Liouville matrix elements for a specific model system. This problem can be resolved by making phenomenological assumptions or treating the unknowns as fitting parameters. However, for the Hamiltonian approach, one encounters a fundamental difficulty in obtaining a complete set of basis vectors at the beginning of the calculation and therefore progress is blocked. The DOS in the leads are captured by the lead DOS functions Γ^*L*^ and Γ^*R*^ which enter via 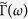 and *ρ*_*d*_(*ω*) in equation (2). Hence, these functions are inputs to the theory, and can be chosen to represent various different scenarios.

We now describe the coherent tunneling processes which produce the dominant features of the experimental *dI*/*dV* curves. Our central claim is that many-body singlet states form between electrons on the MS and in both leads (as shown by the ellipses in Figs 1, 2 and 3). When a bias voltage is applied, the coherent singlet cotunnels unidirectionally through the MS. In this context, coherence implies that the singlet may perform spin exchange or change its partner in the leads without any energy cost. A mesoscopic Kondo system is sketched in Fig. 1a and consists of two leads with constant DOS and a central zero-dimensional MS. This system can be described by a two-reservoir Anderson impurity model^10^. To understand the coherent tunneling mechanism of the entangled state, we must examine the elements of the Liouville operator shown in equation (1). These are illustrated schematically in Fig. 2 and their full operator expressions are given in equation (S6) in the Supplementary Information. To put this discussion in context, Fig. 1b shows an archetypal plot of the tunneling conductance as a function of bias voltage *V* for a quantum point contact (QPC) with symmetric coupling to the leads on both sides^11^. Notice there are three coherent peaks: one “zero bias peak”, and two “side peaks” which cannot be Coulomb peaks because of the small energy scale. We shall demonstrate which of the *γ* elements contribute to the formation of these peaks. We first discuss the formation of the side peaks. Figure 2a and its mirror image 2b show coherent current formed solely from singlet hopping. In this process, a left singlet performs a singlet hop to the right (second sketch) before partner exchange on the left (third sketch) enables a second singlet hop to the right which returns the MS to its original state (fourth sketch). This coherent process allows two electrons (highlighted in yellow in the sketch) to tunnel from the left lead to the right lead without paying the on-site Coulomb repulsion energy cost *U*. Matrix elements 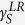 and 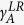 in equation (1) are given by symmetric and antisymmetric combinations 
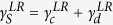
 and 
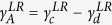
. It is clear that at equilibrium (i.e., when no bias is applied across the system), 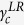 and 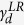 will have the same magnitude because the singlet hops will occur with equal probability in each direction, so that 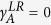 and 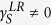, indicating that 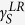 contributes to the zero bias peak. However, out of equilibrium, either 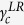 or 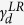 will be suppressed because it must work against the external bias in the system so that 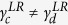 and as a result, 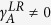. This contributes an additional channel for current at finite bias, and this manifests as the side peaks in *dI*/*dV* away from equilibrium. Therefore, it is the inclusion of left-right antisymmetric coherent superpositions 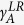 which allow us to explain the existence of the side peaks in the tunneling conductance. Additionally, 
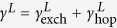
, where 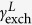 (Fig. 2c) represents the Kondo coupling strength on the left side, and 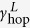 (Fig. 2d) describes the current flow forming the zero bias peak, in which spin exchange is involved. However, the zero bias peak is suppressed in the 2D SCMs because of the interaction-induced gap at the Fermi level and so we do not focus on it in this study. We briefly mention that the *γ* elements are not strongly temperature dependent unless the temperature gets so high that thermal fluctuations destroy the coherence. In fact, the main thermal effects will be driven by the changes in the Fermi-Dirac distribution of the leads, and changes in the sample DOS itself.

In real STS experiments for cuprate superconductors, every copper atom has strong on-site repulsion *U* (shown in Fig. 3a). We set up our model for 2D cuprate superconductors by stating that the tunneling current coming from the STS tip only enters through one copper atom. Then, the tunneling mechanism depends on the strong *U* at that site only so that the rest of the sample can then be modeled as a system of non-interacting Bogoliubov quasiparticles^1^,^12^,^13^ excited by applied bias, as sketched in Fig. 3b. Here, an “entangled state” comprising a linear combination of two singlets (solid and dashed purple loops) accompanied by other coherent spins in the tip (blue and red dots) and the sample is formed. The Hamiltonian for the left lead is chosen to be appropriate for the metallic tip so that Γ^*L*^ is constant, the Hamiltonian for the right lead is taken to be a system of Bogoliubov quasiparticles^1^,^12^,^13^ whose frequency-dependent DOS characterizes the superconductor. Therefore, the DOS of the superconductor becomes an input to the theory [Γ^*R*^(*ω*) in equations (1) and (2)] and hence we can use it as a fitting function to distinguish between various theoretical propoals for the superconductor DOS. This allows us to separate the physics of the tunneling mechanism by coherent co-tunneling of singlet states through the MS from the physics of the superconductor in the bulk of the sample.

Now, we apply the theory described above to correlated superconductors to show that it gives accurate predictions for published STS data and that the features in the sample DOS lead to the fine structure in the low-bias region. We require a phenomenological frequency-dependent sample DOS to use as Γ^*R*^(*ω*). We find that a DOS characteristic of a *d*-wave superconducting order parameter for the under-doped (UD) cuprate and a DOS composed of an *s*-wave gap and a sharp DOS barrier for the pnictide give a well-fitting tunneling conductance. In both cases, we use flat DOS in the high frequency region to emphasize that the side peaks are not created by features in the sample DOS. These DOS functions are shown as the green lines (right-hand axis) in Fig. 4a,b. Choosing the parameters given in the second and third row of Table 1 gives the fit (blue line) to the experimental data (red line). The quantitative agreement up to the side peaks is almost exact, and we reproduce all the low-bias features. The peaks of the DOS are located at Δ_*p*_ = 17.8 meV (Fig. 4a) and Δ_*p*_ = 2.3 meV (Fig. 4b), which are consistent with the superconducting gap reported in recent ARPES data^2^,^7^ and correspond to the low-energy shoulders in the *dI*/*dV*. The result is natural since the Bogoliubov quasiparticles introduced in Fig. 3b describe the coherent superconducting state^1^. We also show the *dI*/*dV* and the DOS for optimally doped (OP) and highly overdoped (HOD) cases in the insets of Fig. 4a. It is noteworthy that the two side peaks in the UD and OP cases are formed in the region of flat DOS. This demonstrates that the side peaks are not connected to the superconducting gap, and they must be reinterpreted as a coherent peak of the non-equilibrium transport mechanism. Their sustained presence when *T* > *T*_*c*_ signifies the existence of pre-formed Cooper pairs^12^ up to T* of cuprate superconductors. Since a big gap observed in the anti-nodal region in the ARPES measurements is not a coherent gap^1^,^2^, the anti-nodal gap is not included in this study. A big difference occurs in the HOD case where strong correlation no longer persists. We obtain the HOD line shape using vanishing *γ* elements for a very weak effective Coulomb interaction. Note that two peaks occur within the *d*-wave region.

In conclusion, we have generalised a non-equilibrium quantum transport theory which accurately models tunneling conductance measurements for extended 2D strongly correlated systems. This theory is therefore crucial in understanding experimental data in a wide variety of different contexts. A particularly interesting point is that our theoretical tunneling conductance and sample DOS for cuprate and pnictide superconductors are consistent with the results obtained by two different experimental techniques. This fact eliminates the temptation to think that the *dI*/*dV* curve is simply a reflection of the sample DOS (as it would be in the weakly interacting case), and therefore to interpret the two side peaks as evidence of a correlated band gap in the sample DOS. We demonstrate that the correct interpretation is that the side peaks are evidence of the coherent non-equilibrium tunneling mechanism.

## Methods

After the appropriate modelling described in the text, the STS experiment may be described by the following Hamiltonian:


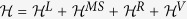


where 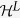 and 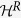 correspond to the left and right leads, which are taken to be a metallic tip and a system of non-interacting Bogoliubov quasiparticles (described in detail in Section III of the Supplementary Information) excited by the applied bias at *T* = 0 K, respectively. The Hamiltonian for the MS is 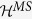, and 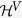 describes the tunneling between the MS and the leads. As described in the Supplementary Information, sections IB and IC, the Liouville operator describing the dynamics of the MS is


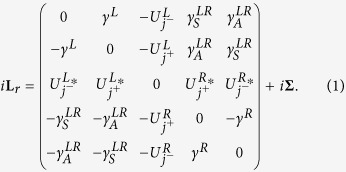


where *i*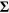 denotes the self-energy matrix with elements 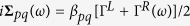. The coefficients *β*_*pq*_ come from the process of matrix reduction which generates equation (1). Each matrix element has its own unique role in determining *dI*/*dV*. The middle row and column of the matrix are the incoherent couplings of the MS to the leads which represent double occupancy at the MS via the fluctuations of incoherent spins from the tip or the sample. Therefore, these elements represent the effective Coulomb interaction. The doping effect is given by the imaginary part of these elements shown in equation (S7). The subscript *j*^−^ indicates the current operator, whereas *j*^+^ indicates the sum of the left and right movements.

Within this formalism, the tunneling conductance at zero temperature can be obtained as discussed in the Supplementary Information, sections IB and IE:


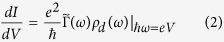


where 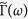 is the effective coupling of the left lead, the MS, and the right lead and is given by


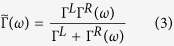


for 2D SCMs. The quantity 
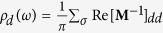
, where the matrix **M** is given by the elements 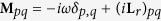, is the non-equilibrium local DOS at the MS (which is distinct from the sample DOS) and depends on the strong interactions. It is computed from the Liouville matrix in equation (1) as described in the Supplementary Information.

## Additional Information

**How to cite this article**: Hong, J. and Abergel, D. S. L. A universal explanation of tunneling conductance in exotic superconductors. *Sci. Rep.* **6**, 31352; doi: 10.1038/srep31352 (2016).

## Figures and Tables

**Figure 1:**
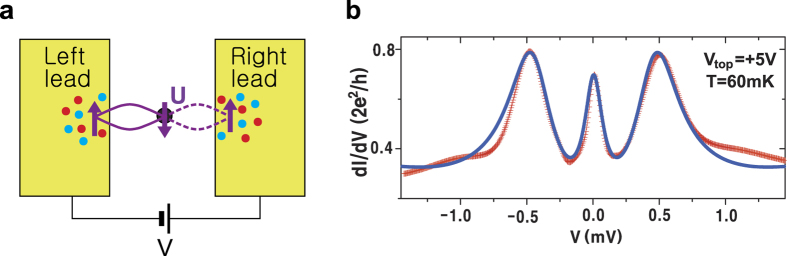
A schematic of the mesoscopic Kondo system and typical experimental *dI*/*dV* data. (**a**) An entangled state is composed of a linear combination of singlets (the solid and dashed magenta loops) and coherent up- and down-spins denoted by different colored dots. (**b**) Tunneling conductance for a QPC. Experimental data (red) reported in ref. 11, theoretical results (blue) obtained with parameters in the first line of Table 1.

**Figure 2:**
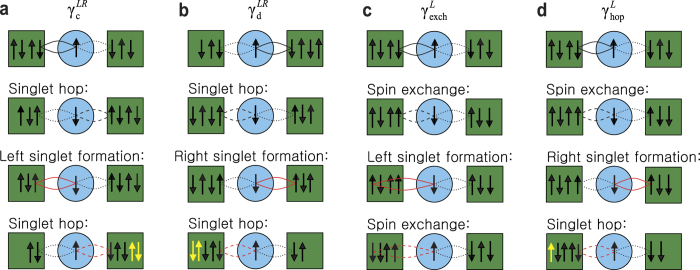
Coherent hybridization dynamics of *γ*. The first row indicates the initial entangled states before performing hybridization dynamics (subsequent rows). The black dotted line is added to represent an entangled state. The dashed line indicates the state after exchange or singlet hopping, and the red solid line indicates the singlet formation for next hybridization process. The yellow arrows indicate transferred spins. (**a**,**b**) Finite-bias coherent current formation in 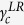 and 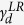 exclusively by singlet hopping. (**c**) The exchange process contribution to *γ*^*L*^. (**d**) Low-bias coherent current forming dynamics in *γ*^*L*^ which is given by the combination of spin exchange and singlet hopping.

**Figure 3:**
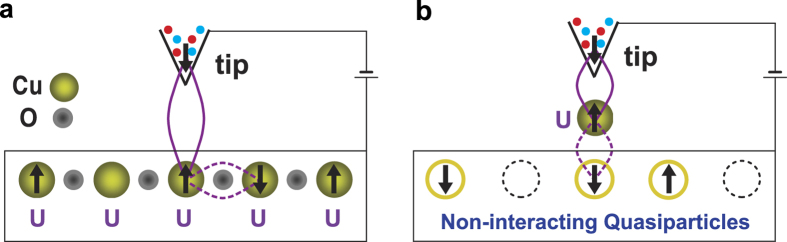
Schematics of STS experiments on 2D cuprate superconductors. (**a**) A schematic of an STS experiment for a real cuprate superconductor. All Cu atoms have on-site repulsion *U* and the MS is located just below the tip. (**b**) A schematic of the model from the many-body point of view. There is a single MS with on-site repulsion *U*, and the lower part is a non-interacting system of Bogoliubov quasiparticles excited by applied bias, whose DOS is that of a correlated superconductor.

**Figure 4:**
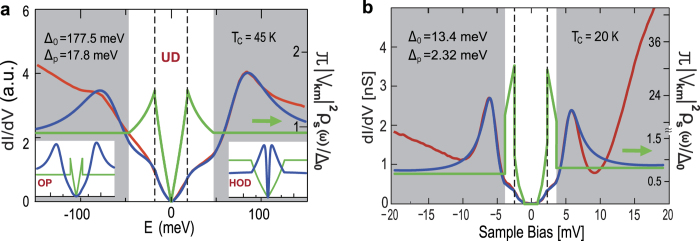
Comparison with experiment: Correlated superconductors. In each case, the red line is experimental data, the blue line is the prediction of our theory, and the green line (right axis) is the phenomenological sample DOS used to obtain the fit. The vertical dashed line indicates the edge of the shoulder corresponding to the peak in the sample DOS. The grey shading denotes the region of flat sample DOS. (**a**) STS data of cuprate superconductor Bi_2_Sr_2_CaCu_2_O_8+*δ*_ given in ref. 3. Insets: OP (left) and HOD (right) cases. (**b**) STS data of pnictide superconductor LiFeAs given in ref. 6.

**Table 1:** Values of matrix elements and *β*.

Figure	*γ*^*L*^	*γ*^*R*^	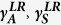	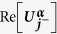	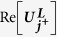	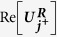	Im[*U*^*α*^]	Γ^*L*^	Δ_0_
1b	0.53	0.53	0.68	1.0	1.17	1.17	0	0.8	0.99
4a	0.01	0.5	0.5	1.75	0.35	2.45	0.1	0.24	118
4b	0.01	0.5	0.5	3.5	0.7	3.85	0	0.24	13.4

The values of the matrix elements appearing in equation (1) used to create the figures. All values are in units of Δ_0_, except Δ_0_ (meV). Note that the value in the Γ^*L*^ column for Fig. 1c is actually 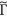.

*β*_11_ = *β*_15_ = *β*_55_ = 0.252, *β*_12_ = *β*_14_ = *β*_25_ = *β*_45_ = 0.254, *β*_22_ = *β*_24_ = *β*_44_ = 0.258,

*β*_33_ = 1, and *β*_13_ = *β*_23_ = *β*_43_ = *β*_53_ = 0.
